# Supplementary figures and images for: Persistence of phenotypic responses to short-term heat stress in the tabletop coral Acropora hyacinthus
Source: PLoS One. 2022 Sep 9;17(9):e0269206. doi: 10.1371/journal.pone.0269206 (PMC9462741; doi:10.1371/journal.pone.0269206)

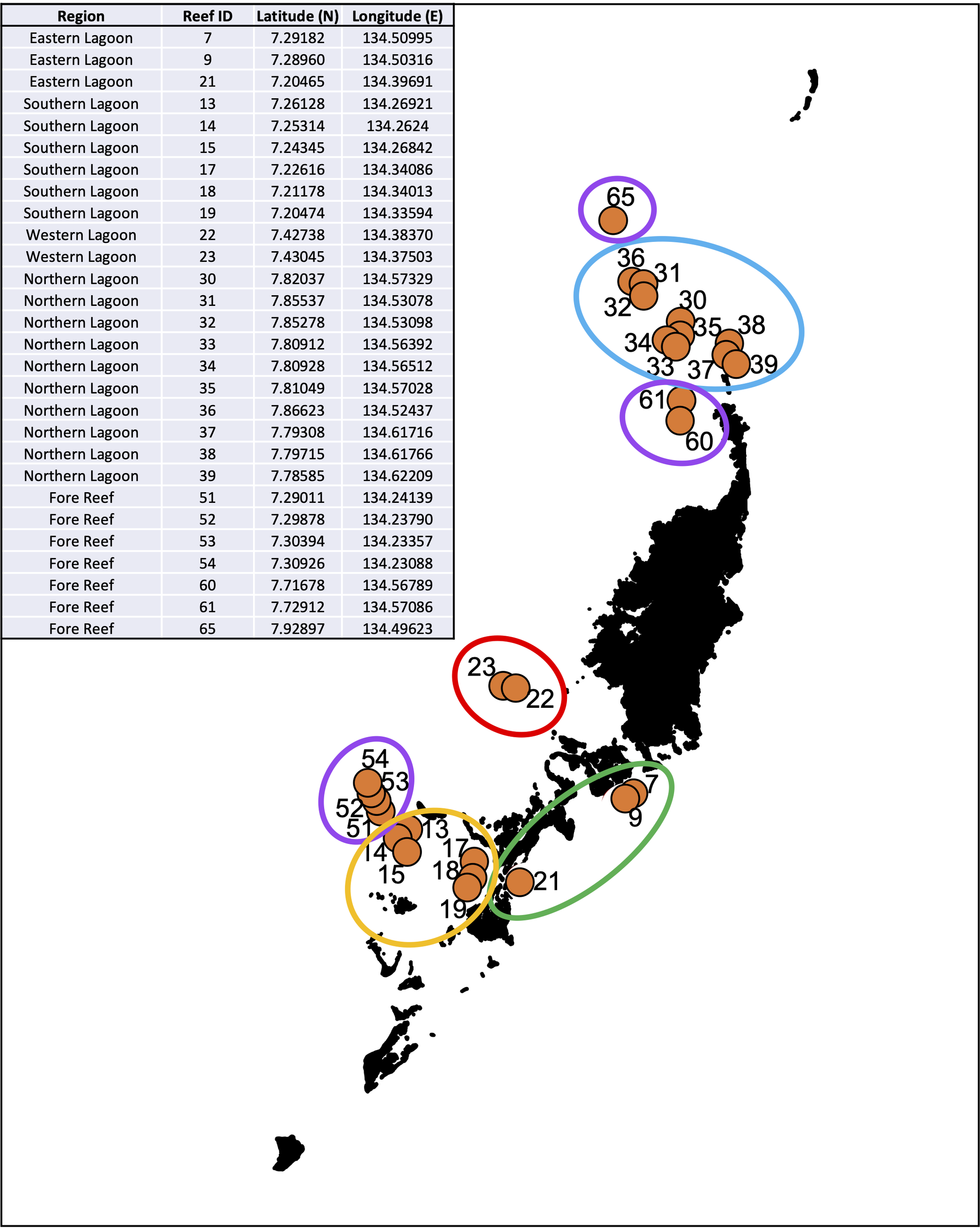

Supplement: S1 Fig — Map of Palau with relative reef positions, created using R packages rgeos, mapdata, and rgdal and the “Palau_Shoreline" shapefile from USGS. Reefs are represented by orange circles and grouped based on reef clusters; patch reefs are circled in red, yellow, green, and blue according to their region, and fore reef sites are circled in purple. Latitudinal and longitudinal coordinates of all reefs are included and written in decimal degrees. (TIF) [file pone.0269206.s001.tif]

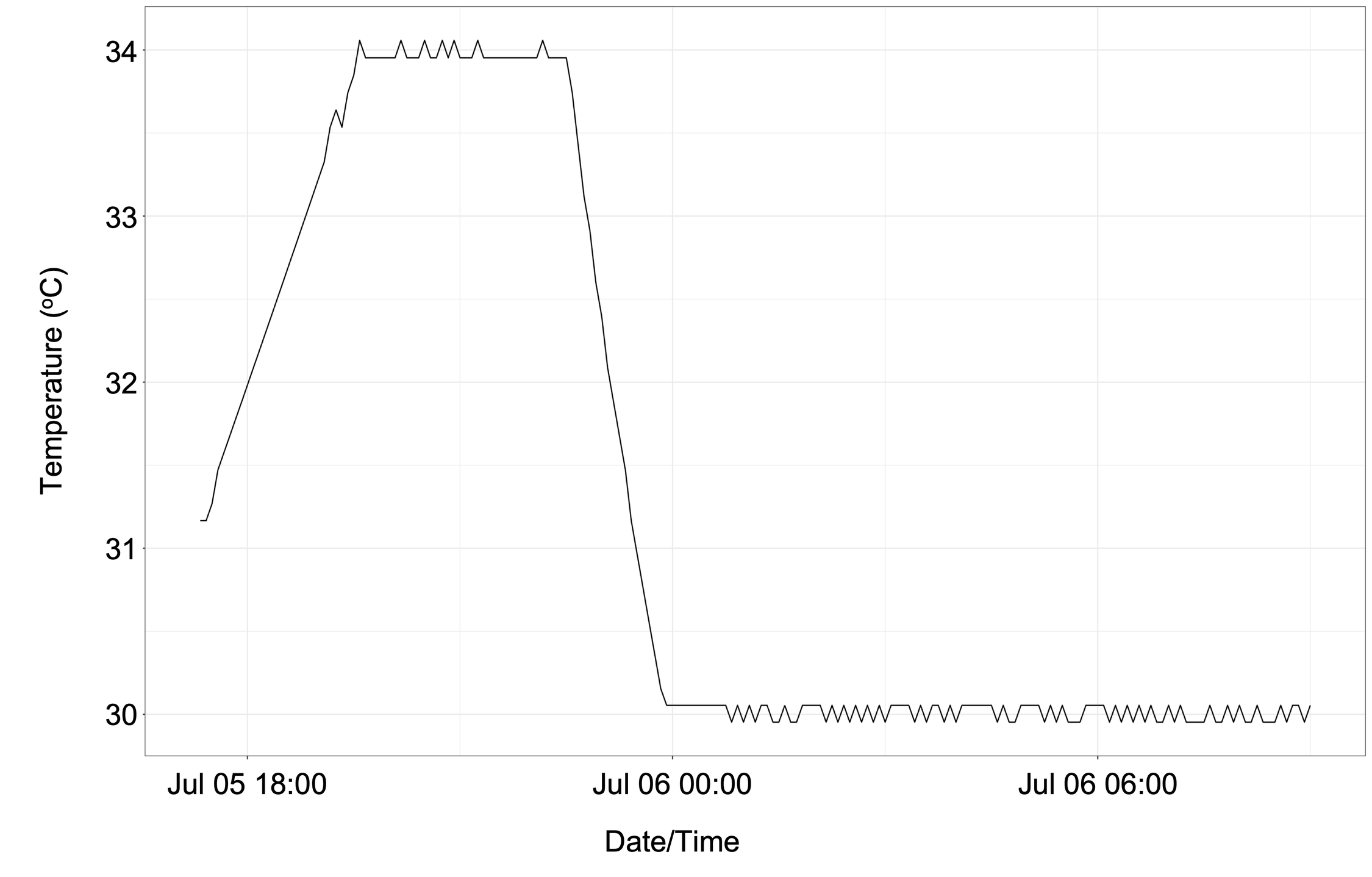

Supplement: S2 Fig — Representation of the temperature ramp system in a heat stress tank outfitted with a heater, chillers, a light fixture, and water inflow and outflow tubing as described further in the methods section. Real-time temperature measurements were collected using a HOBO logger recording temperature in 10-minute intervals. This example temperature ramp was measured on July 5th 17:20 until July 6th 09:00 2022. The ramp up period (31–34°C) was programmed for 2.5 hours, the hold period (34°C) was programmed for 2.5 hours, and then the ramp down and hold at 30°C was programmed for 10 hours and 40 minutes. Note that this example temperature ramp system differed from the ramp system conducted for analysis (3 hour ramp up from 30 to 34.5°C, 3 hour hold at 34.5°C, and ramp down to 30°C) but these measurements aimed to demonstrate the high degree of accuracy and precision of the temperature controller system. (TIF) [file pone.0269206.s002.tif]

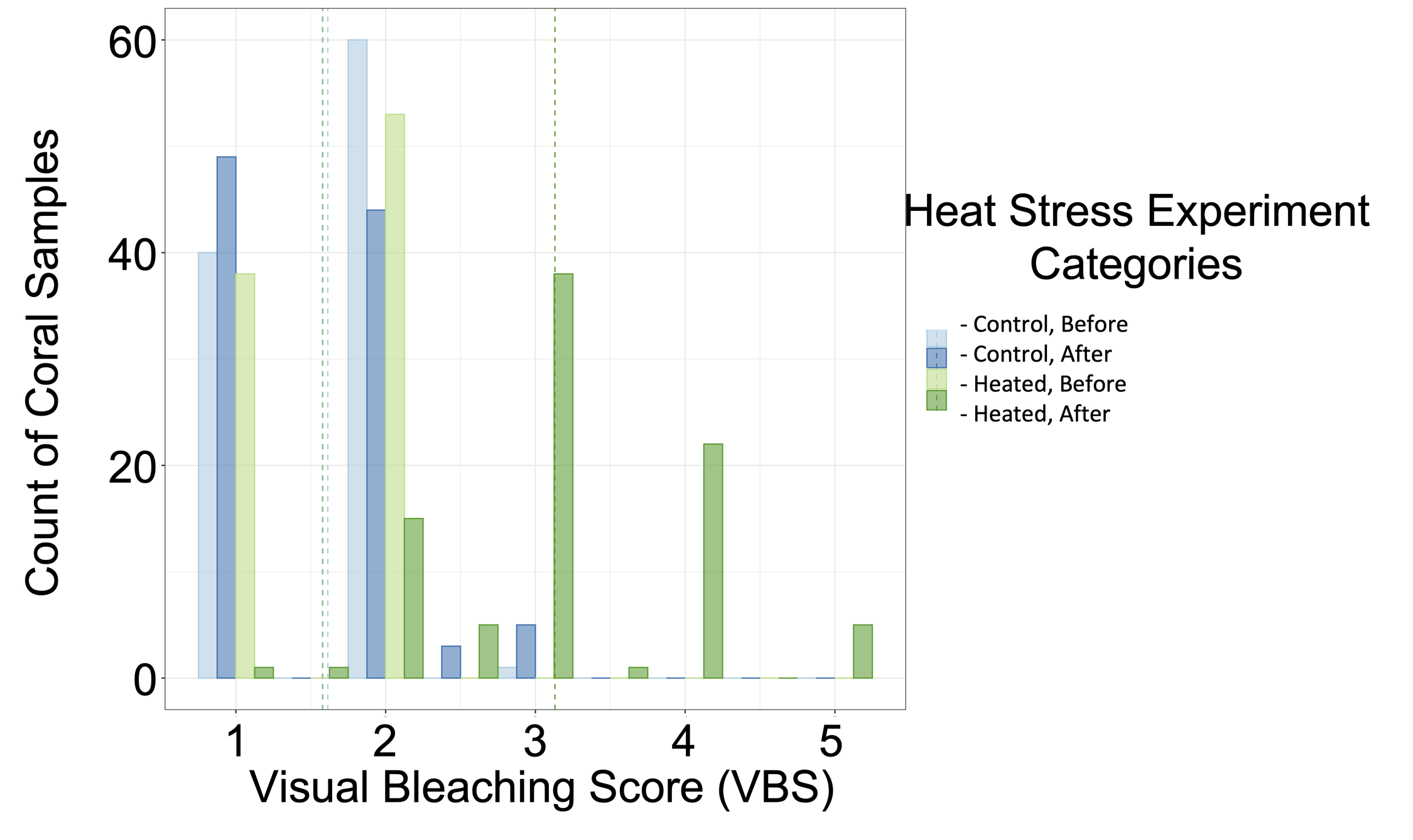

Supplement: S3 Fig — Counts of control (blue) and bleached (green) colony samples before and after the two-day short-term heat stress experiment. Bleaching severity before and after heat stress was measured by visual bleaching score (VBS). (TIF) [file pone.0269206.s003.tif]

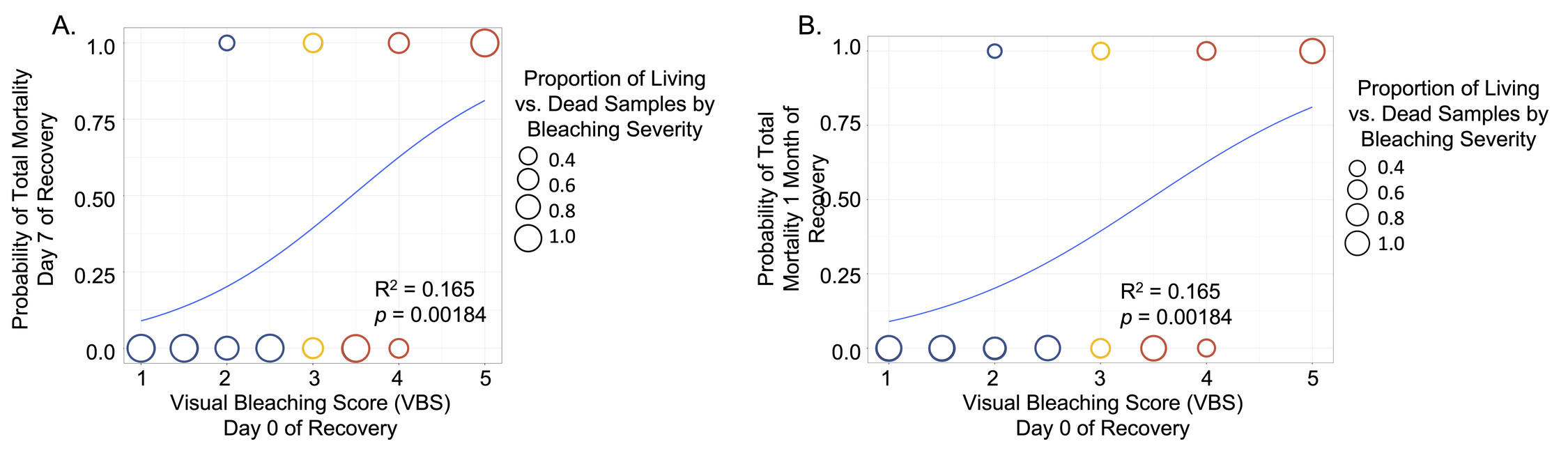

Supplement: S4 Fig — Binomial representation of living (value = 0) and dead (value = 1) coral fragments 7 days (A) and 1 month (B) after heat stress versus bleaching severity on Day 0 post-stress, where circle sizes represent proportion of living vs. dead samples at each VBS category (mixed effects logistic regression, accounting for spatial variability, with pseudo R2 values provided). Note that identical statistics results from both logistic regressions are due to no corals dying between Day 7 and Month 1 timepoints. Circle colors correspond to bleaching severity immediately after heat stress: blue = low bleaching, yellow = moderate bleaching, and red = high bleaching. (TIF) [file pone.0269206.s004.tif]

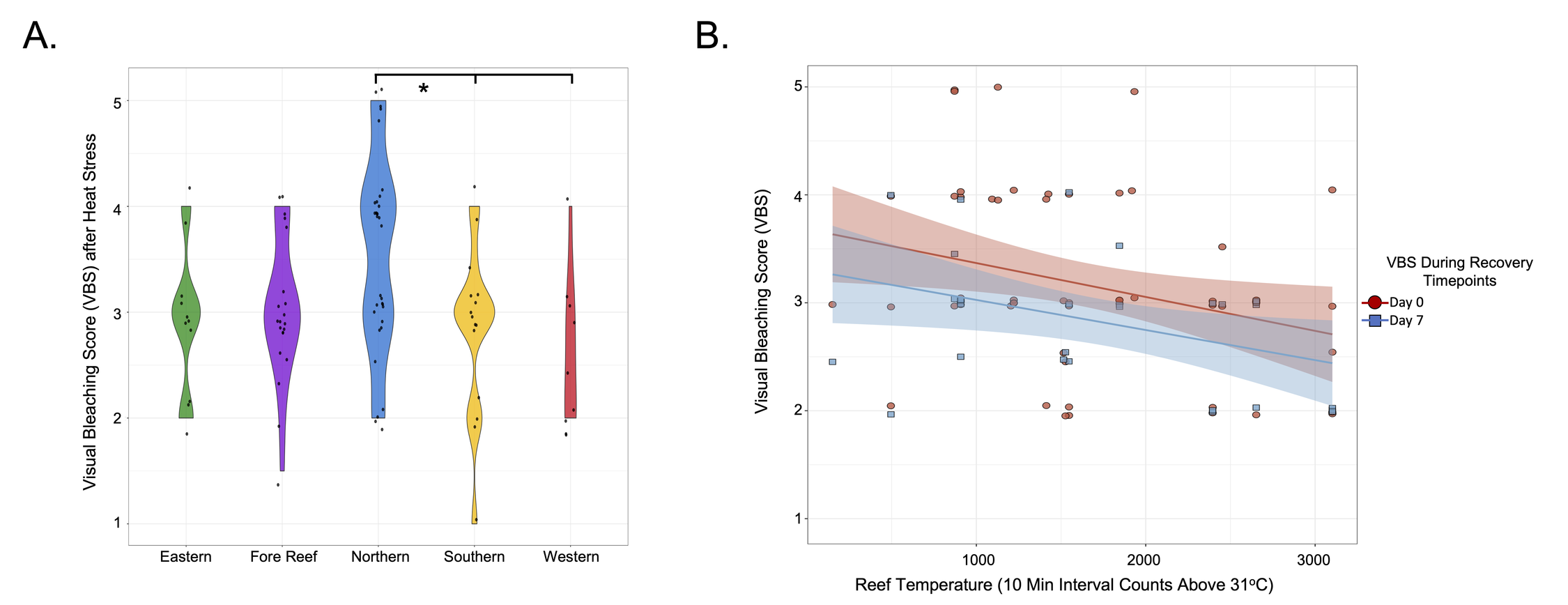

Supplement: S5 Fig — (A) Violin plot of bleaching severity and geographic locations. Bleaching severity is measured through visual bleaching scores (VBS 1, no bleaching, to VBS 5, total bleaching), and each point denotes a fragment that represents a coral colony within geographic locations. All locations are in lagoons apart from the Fore Reef category, and colors correspond to groups in S1 Fig. We ran an ANOVA and Tukey test for significance (S2 Table), * denotes p ≤ 0.05. (B) Scatterplot showing the relationship between reef temperature extremes (represented by 10-minute interval HOBO logger counts above 31°C) and visual bleaching score with 95% confidence intervals. Day 0 and 7 after heat stress are shown. Linear regression results: Day 0, p = 0.02167, R2 = 0.0731, df = 57, and Day 7, p = 0.0292, R2 = 0.1287, df = 28. (TIF) [file pone.0269206.s005.tif]

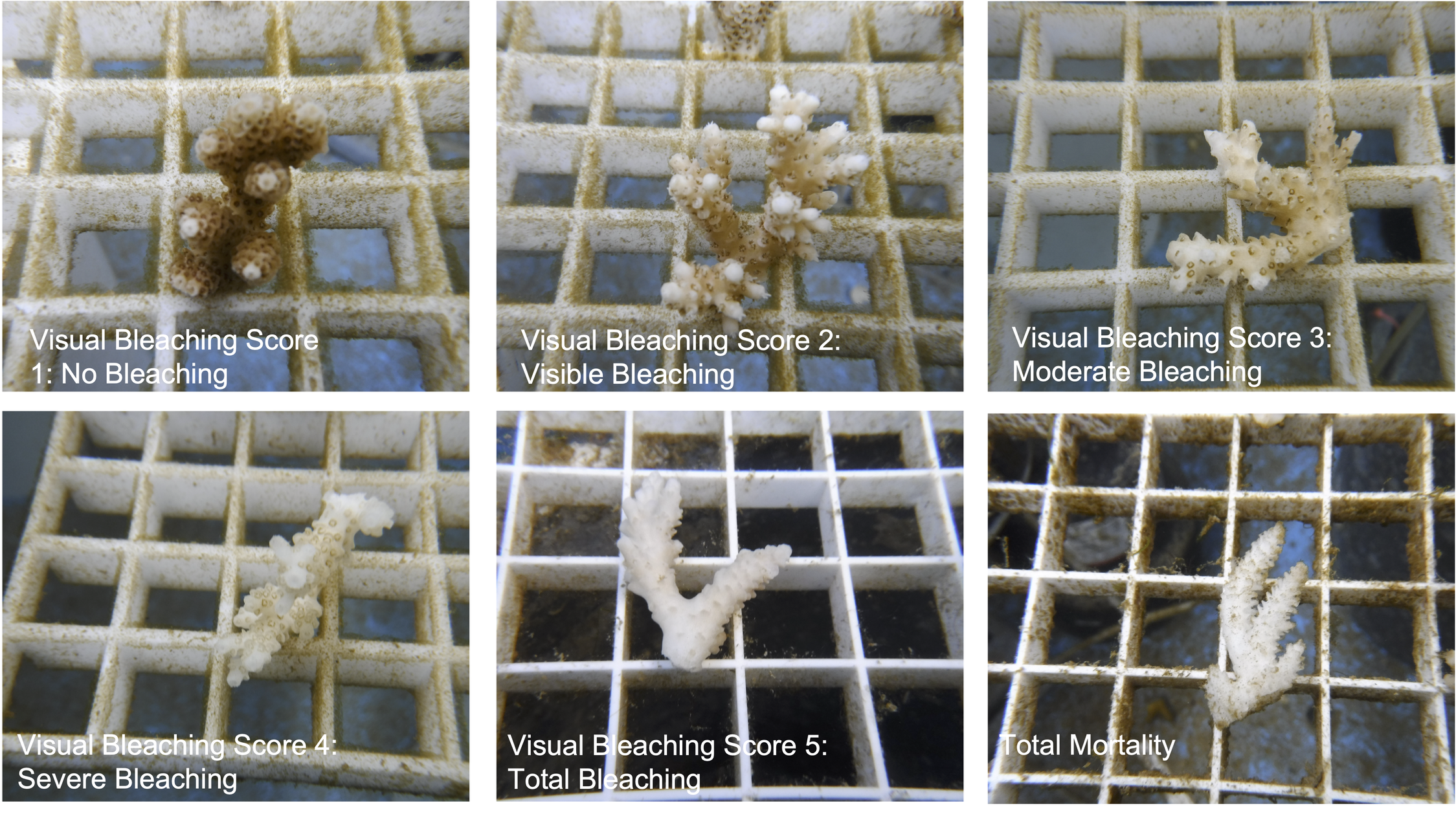

Supplement: S6 Fig — Two observers determined each fragment’s visual bleaching score category and mortality. Here, one fragment example is provided per visual bleaching score category and mortality category. (TIF) [file pone.0269206.s006.tif]
